# Evaluating Traditional Bonesetting Interventions: A Methodological Review and Framework for Clinical Research

**DOI:** 10.7759/cureus.116532

**Published:** 2026-09-19

**Authors:** Jin Ai Hua, Ba Hu Shan, Ta Na, Wu Da Mu, Deng Yu Qi, Tsend-Ayush Damba, Bolortulga Zagdsuren, Dariimaa Ganbat

**Affiliations:** 1 Traditional Therapy, International School of Mongolian Medicine, Mongolian National University of Medical Sciences, Ulaanbaatar, MNG; 2 Scientific Research, Ulanqab Integrated Hospital of Traditional Chinese and Mongolian Medicine, Ulanqab City, CHN; 3 Administration, International School of Mongolian Medicine, Mongolian National University of Medical Sciences, Ulaanbaatar, MNG; 4 Medical Science, Mongolian National University of Medical Sciences, Ulaanbaatar, MNG

**Keywords:** clinical epidemiology, comparative effectiveness, complex interventions, fracture care, intervention reporting, patient safety, traditional bonesetting, traditional bone-setting

## Abstract

Traditional bonesetting (TBS) remains an important source of fracture care in many low- and middle-income settings. Still, its evidence base is difficult to interpret because care pathways are heterogeneous, practitioner-dependent, and often incompletely specified. This methodological review synthesizes recurring design and reporting problems in TBS research and translates established complex-intervention principles into a practical framework. PubMed was the principal biomedical source, supplemented by Google Scholar, citation chaining, and targeted searches of methodological and implementation literature; the literature update extended through August 31, 2026. Major threats to interpretation include exposure misclassification, poorly described provider expertise and comparators, confounding by indication, absent pretreatment outcomes, inconsistent follow-up, passive harm ascertainment, and limited fidelity assessment. Recent observational evidence linking specific immobilization and topical practices to serious outcomes, along with training and collaborative-care studies, reinforces the need to evaluate components and care pathways rather than the TBS label alone. We propose the TBS-8 Framework: intervention specification; provider characterization, training, and expertise; comparator definition; baseline prognostic measurement; allocation and confounding control; outcome and follow-up architecture; active safety surveillance; and process evaluation. Randomized and pragmatic designs are appropriate only when genuine clinical equipoise exists and randomization is ethical and feasible; prospective comparative cohorts can be informative when preference, access, or safety considerations limit randomization. The TBS-8 Framework is conceptual and unvalidated. Its purpose is to translate established methodological principles and recurrent TBS-specific problems into eight proposed core domains for reproducible, measurable, and safety-conscious comparative-effectiveness and implementation research.

## Introduction and background

Traditional bonesetting (TBS) encompasses diverse fracture-care practices that remain active across parts of Africa and Asia. The label is clinically broad: reported practices may include manual reduction, traction, external splinting, topical or herbal applications, massage, staged mobilization, and other culturally specific components. The intervention is therefore better understood as a complex health intervention than as a single technical procedure. Complex interventions contain interacting components, vary across contexts, and may depend strongly on provider skill, recipient characteristics, and implementation conditions [1,2]. These characteristics are directly relevant to TBS, for which the same treatment label may conceal materially different care packages.

TBS continues to be used despite the availability of modern orthopedic services in many settings. Reasons described in the literature include cultural trust, geographic accessibility, perceived affordability, family influence, dissatisfaction with hospital care, and fear of surgery or amputation [3-6]. These determinants matter methodologically because they also influence prognosis and treatment selection. A patient who chooses TBS may differ from a patient who presents directly to an orthopedic service in injury severity, delay to care, socioeconomic resources, health beliefs, access to transport, willingness to undergo surgery, and capacity for follow-up. Consequently, crude comparisons between TBS and hospital care can reflect differences in who receives each treatment rather than the treatment itself.

Safety is a central concern. Two systematic reviews of TBS complications in low- and middle-income countries found that the available literature is dominated by observational and hospital-based studies and documented substantial morbidity, including malunion, nonunion, infection, joint stiffness, gangrene, and amputation [7,8]. Individual series have similarly described severe complications after prior TBS treatment [9-12]. These reports establish an important safety signal, but they also reveal a fundamental evidence problem: many studies begin only after patients with complications arrive at a hospital. Such designs are useful for characterizing harms among referred patients, but they cannot estimate the incidence of complications among all people treated by TBS or establish comparative effectiveness.

At the same time, the evidence base is not static. Prospective cohort work has attempted to compare outcomes between fracture patients who did and did not visit traditional bonesetters [13], and recent Mongolian medicine research has used repeated clinical and biological measurements to evaluate specific components of traditional fracture management [14]. These developments increase the need for a common methodological language. Without clear intervention definitions, provider descriptions, baseline measurements, comparator protocols, longitudinal outcome schedules, and active adverse-event surveillance, more studies will not necessarily produce more interpretable evidence.

Generic guidance for complex interventions already exists, and this review does not attempt to replace it. Template for Intervention Description and Replication (TIDieR) is principally concerned with sufficiently complete intervention description for replication, while contemporary complex-intervention guidance emphasizes context, mechanisms, implementation, and selection of designs that match the research question. The methodological gap addressed here is translational: TBS research still requires a single scaffold that brings those general principles together around recurrent field-specific problems, including component-level heterogeneity, non-formal and variable provider training pathways, heterogeneous comparators, treatment preference and delayed referral, incomplete treatment denominators, and passive ascertainment of harms. Investigators also need TBS-specific decisions about how to describe a locally manufactured splint so that another team can reproduce it, how to characterize a practitioner whose expertise may derive from apprenticeship or community recognition rather than formal certification, how to define a comparator when the alternative pathway is itself heterogeneous, how to measure pretreatment prognosis when treatment preference shapes allocation, and how to count harms in patients who may never reach a hospital. The purpose of this review is therefore methodological rather than promotional or dismissive. We ask how TBS should be evaluated so that future studies can generate evidence that is reproducible, clinically meaningful, and capable of informing patient safety, comparative effectiveness, practitioner training, and health-system decisions. We synthesize recurring weaknesses in the published literature and propose an eight-domain framework for designing, analyzing, and reporting future TBS research [15-23].

## Review

Literature identification and analytical approach

This article is a purposive methodological literature review rather than a formal systematic or scoping review. That classification is deliberate. The source base includes heterogeneous clinical studies, systematic reviews, qualitative and practitioner-focused studies, and methodological guidance, while the purpose is conceptual synthesis rather than exhaustive effect estimation. We therefore do not claim complete evidence capture, report pooled treatment effects, present study-count percentages as if they were derived from protocolized screening, or perform a formal risk-of-bias assessment. The included studies are used to identify and illustrate recurring methodological problems relevant to TBS research.

PubMed was used as the principal biomedical source, supplemented by Google Scholar, backward and forward citation chaining, and targeted searches for complex-intervention, comparative-effectiveness, causal-inference, training, referral, and implementation literature. A targeted literature update was completed on August 31, 2026. Search concepts included traditional bonesetter/bonesetting, fracture care, complications and safety, training and referral, intervention reporting, complex interventions, pragmatic trials, and observational causal inference. Zotero was used to organize references. Source selection was purposive and iterative and was conducted through author-team review and discussion rather than independent duplicate screening. Candidate sources were retained when they materially informed at least one methodological domain or supplied particularly informative contemporary clinical, training, referral, or collaborative-care evidence. No protocolized language restriction was imposed in the search logic; however, selection depended on sources the author team could reliably interpret, so relevant non-English evidence may have been missed. Peer-reviewed primary clinical studies, systematic reviews, practitioner/collaboration studies, and established methodological guidance were prioritized; preprints and non-indexed material were not used as primary clinical evidence when a citable peer-reviewed source was available.

The studies in Table 1 were purposively selected to illustrate complementary methodological problems across study designs, patient populations, geographic settings, and contemporary evidence rather than to represent a comprehensive or quality-ranked sample.

**Table 1:** Selected illustrative evidence for methodological challenges in traditional bonesetting research TBS: traditional bonesetting. Studies were purposively selected to illustrate complementary methodological problems across study designs, populations, geographic settings, and contemporary evidence. The table is illustrative rather than comprehensive, statistically representative, or quality-ranked.

Study	Design / setting	Methodological contribution	Key limitation for causal interpretation
Onuminya, 2004 [3]	Descriptive evaluation of primary fracture care in Nigeria	Detailed description of practice and the role of TBS in primary fracture care	Not designed to estimate comparative treatment effects
Nwachukwu et al., 2011 [4]	Qualitative and contextual comparison of traditional and orthopedic fracture care	Illustrates TBS as a multi-component, context-dependent care pathway	Primarily descriptive; intervention fidelity and standardized outcomes were not central
Dada et al., 2009 [9]	Prospective hospital series of patients presenting with TBS-related complications	Demonstrates clinically important complications and reasons for patronage	Denominator is patients presenting with complications, not all TBS-treated patients
Adem et al., 2025 [12]	Multicenter prospective study of children presenting to eight hospitals after TBS	Links specific treatment components, rather than the TBS label, to amputation and prolonged hospitalization	Hospital-presenting sample; component exposures are self-reported and not randomly assigned
Yimenu and Mengist, 2022 [13]	Prospective cohort of fracture patients with and without prior TBS exposure	Shows feasibility of prospective comparative follow-up and multivariable analysis	Nonrandom treatment selection leaves potential residual confounding
Xie et al., 2026 [14]	Comparative Mongolian medicine study with repeated functional and biomarker assessments	Illustrates component-specific and longitudinal evaluation of a defined intervention	Single-setting study; generalizability and replication depend on detailed intervention and allocation reporting
Onyemaechi et al., 2021 [7]	Systematic review of complications in low- and middle-income countries	Demonstrates broad burden of reported morbidity and hospital-based evidence concentration	Primary evidence is observational and frequently based on referral populations
Garikapati et al., 2023 [8]	Systematic review of TBS-associated complications	Confirms recurrent severe complications across multiple settings	Does not resolve incidence among all treated patients or comparative effectiveness

The appendix provides the literature-identification approach and reproducible example search strings. This approach supports transparency and broad reproducibility of the literature-identification logic but, by design, does not permit exact independent reproduction of the final source set. The synthesis focuses on research design, measurement, safety, and reporting rather than the direction or magnitude of treatment effects.

The analytical framework was informed by the TIDieR, which emphasizes complete reporting of intervention content, provider, delivery, dose and timing, tailoring, modification, and fidelity [15]. Contemporary complex-intervention guidance additionally emphasizes context, mechanisms, implementation, and alignment of design with the causal question [2,16-18,22,23]. TBS-8 does not replace these frameworks. Instead, it assembles their general principles around recurrent TBS-specific problems: component-level heterogeneity in manipulation, splinting and topical treatments; variable and often non-formal provider training pathways; heterogeneous comparators; strong treatment preference and delayed referral; hospital-based complication denominators; and the need for active safety and referral capture. Thus, intervention specification and process/fidelity domains are most directly adapted from TIDieR and complex-intervention guidance, whereas the emphasis on provider characterization, pretreatment prognosis, allocation/confounding, defined treatment denominators, active safety surveillance, and referral/escalation reflects recurring interpretive problems in the TBS literature.

Traditional bonesetting as a complex intervention

The first methodological error in this field is to treat "traditional bonesetting" as a sufficiently precise intervention label. In the literature, TBS may include manual manipulation or reduction, traction, tight or semi-rigid splints made from locally available materials, topical preparations, massage, scarification, immobilization, progressive mobilization, spiritual practices, or combinations of these elements [3-5]. Even within a single region, techniques may vary between practitioners and may be tailored according to fracture site, swelling, pain, perceived healing stage, or family tradition.

This heterogeneity has two consequences. First, treatment effects cannot be attributed to one component when several are delivered together. Second, the same intervention name may not represent the same treatment across studies. If one cohort receives reduction plus carefully adjusted splinting and progressive rehabilitation while another receives prolonged tight immobilization and repeated manipulation, pooling the two under a single TBS category obscures clinically meaningful differences. The same problem applies to comparator groups: "hospital care" may represent casting, surgery, observation, physiotherapy, or a sequence of these interventions.

For this reason, the unit of evaluation should be the defined care pathway rather than the cultural label alone. Every study should specify the fracture population, treatment components, sequence, timing, provider, dose or frequency, permitted co-interventions, criteria for modification, and treatment fidelity. TIDieR is particularly useful because its logic applies across randomized and observational designs and to both experimental and comparator interventions [15]. 

Intervention specification and reproducibility

A replicable intervention description is the foundation of interpretable TBS research. Published descriptions commonly identify splinting, manipulation, herbal treatment, or massage but omit details that determine treatment dose and mechanical effect. For splinting, relevant variables include the material, padding, limb position, circumferential pressure, joint inclusion, duration of application, frequency of adjustment, and criteria for discontinuation. For manipulation, authors should report the indication, analgesia or anesthesia, imaging, direction and number of attempts, reduction criteria, and post-reduction assessment. Topical preparations require ingredient identification, preparation method, dose, frequency, quality control, and monitoring for skin reactions. Rehabilitation requires timing, exercise type, supervision, progression criteria, and adherence measurement.

The consequences of omitting this detail are not hypothetical. In a multicenter prospective study of children presenting to Ethiopian hospitals after TBS, the odds of amputation and of prolonged hospitalization varied with specific treatment components, including the type of immobilization applied and the class of topical material used, rather than with exposure to TBS as such [12]. Whatever residual confounding remains in a design of this kind, the finding demonstrates the central point of this section: the label carries no risk information, whereas the splint construct and the topical material do. A study that records only the label cannot generate, replicate, or refute a result of this type, and cannot inform a practitioner training intervention that would need to target the specific practice concerned.

The problem is not unique to traditional medicine. Incomplete descriptions of complex nonpharmacological interventions are recognized across health research, which is why TIDieR was developed [15,16]. TBS studies should therefore be held to the same reporting standard as other complex interventions, not to a lower or culturally exceptional standard. Complete reporting protects both scientific interpretation and traditional practice by allowing readers to distinguish well-specified protocols from unsafe or poorly described variants.

Provider characterization, training, expertise, and treatment fidelity

Provider skill is likely to be an important effect modifier in a manual, practitioner-dependent intervention, yet practitioner characteristics are often reported only as "traditional bonesetter" or "healer." Contemporary qualitative work continues to show that training may be acquired through family transmission, apprenticeship, observation, or spiritual traditions, with wide variation in formal education and clinical exposure [5,19]. This variability is not merely sociological context; it is a potential source of outcome heterogeneity.

Future studies should report the number of practitioners; the training pathway or source of expertise (including apprenticeship, family transmission, community recognition, or formal training); years of experience; case volume; fracture types usually managed; formal certification where applicable; and any study-specific preparation. In this review, “provider characterization, training, and expertise” is intentionally broader than formal credentialing because relevant expertise may be acquired and recognized differently across cultural and regulatory settings. The intervention should also include a fidelity plan. Fidelity concerns whether the protocol was delivered as intended; adherence concerns whether patients received or followed the prescribed intervention. Practitioner checklists, treatment logs, standardized photographs of splint application where appropriate, rehabilitation diaries, and periodic protocol review can make these constructs measurable rather than assumed.

Training itself can be evaluated as an intervention. Earlier reports and expert consensus suggested that structured instruction, referral criteria, and collaboration could modify TBS practice [20,21,24]. A Ghanaian proof-of-concept program trained 157 traditional bonesetters and documented improved knowledge, changes in practice, and 37 referrals to local hospitals during six months of follow-up [25]. A Nigerian feasibility study identified willingness among traditional bonesetters and orthopedic surgeons to support formal training [26], while a 2025 collaborative orthopedic trauma course in rural Tanzania demonstrated the feasibility of joint training between formal healthcare workers and traditional bonesetters [27]. Subsequent Tanzanian work extended this approach to a stepped-wedge cluster-randomized pilot, and a systematic review summarized broader collaboration initiatives [28,29]. These studies do not establish equivalence of TBS and modern fracture care, but they show that provider competence, referral behavior, infection-control practice, and collaboration are modifiable components that can be prospectively evaluated.

Comparator definition

Comparative studies are uninterpretable when the comparator is labeled only as "orthodox care," "hospital treatment," or "surgery." A valid comparison requires equivalent detail for both arms. If TBS is described by component but conventional care is not, observed differences cannot be linked to a known care pathway. Conversely, studies of patients presenting to hospital after failed TBS treatment should not treat the hospital episode as a contemporaneous comparator, because it occurs after a different clinical trajectory.

Comparator selection should follow the clinical question. For stable fractures in which several conservative strategies are reasonable, the comparator may be protocolized nonoperative care. For fractures with accepted surgical indications, a surgical comparator may be appropriate only when genuine equipoise exists and ethical safeguards are met. For health-system questions, referral-based or co-management pathways may be more relevant than head-to-head treatment efficacy. Each comparator requires intervention-level description, including provider expertise, rehabilitation, follow-up, and treatment conversion criteria.

Treatment allocation and confounding by indication

Randomization is the most direct way to protect comparative studies from measured and unmeasured baseline confounding, but it is appropriate only when genuine clinical equipoise exists. Randomization to a TBS pathway should not be undertaken when an injury has an established urgent surgical or specialist indication or when delay could reasonably cause harm. Such patients require prompt referral or definitive care rather than trial allocation. When randomization is ethically and clinically appropriate, allocation concealment, prespecified eligibility, urgent-referral and rescue criteria, protocolized crossover or treatment-conversion rules, and intention-to-treat analysis should be reported. Orthopedic methodology recognizes that explanatory and pragmatic trials answer different questions and that trial design should match the intended inference [22].

When randomization is not feasible, observational designs must treat confounding by indication as a central design problem rather than a statistical afterthought. Patient choice of TBS can be associated with injury severity, financial resources, culture, geography, prior hospital experience, and delay to treatment [6,13]. The core requirements are prospective pretreatment measurement of prognostic factors, a prespecified causal rationale for confounder selection, transparent description of treatment allocation, and an analysis appropriate to the design. Depending on the study question, sample size, overlap, and data quality, regression adjustment, propensity-score methods, inverse probability weighting, matching, or doubly robust estimation may be considered as context-dependent options. No statistical method can recover information that was never measured, and advanced adjustment should not be presented as a substitute for careful design [23].

Baseline prognostic measurement

Many TBS complication studies begin after treatment has already occurred. This creates a baseline problem: investigators may know the patient’s status at hospital presentation but not the fracture severity, soft-tissue condition, pain, function, or neurovascular status before TBS treatment. A complication documented later may have arisen from the original injury, from delayed presentation, from the TBS intervention, or from an interaction among these factors.

Future comparative studies should collect a standardized pretreatment minimum dataset. At a minimum, this should include age, sex, relevant comorbidities, injury mechanism, fracture location and classification, displacement or angulation when relevant, open versus closed injury, neurovascular status, time from injury to treatment, prior treatment, baseline pain and function, and treatment preference. Additional context such as distance to care, insurance or ability to pay, and health literacy may be necessary when these variables plausibly influence both treatment selection and outcome.

Outcome architecture and follow-up

The literature historically emphasizes complications and radiographic outcomes, while validated patient-reported and function-oriented measures are less consistently used. Future studies should prespecify a compact outcome set matched to fracture site, age, language, cultural context, research purpose, and locally established measurement properties. Adult examples include the Disabilities of the Arm, Shoulder and Hand (DASH) for upper-limb disability, the Lower Extremity Functional Scale (LEFS) for lower-limb function, the EQ-5D-5L for health-related quality of life, and a visual analog scale (VAS) or numerical rating scale (NRS) for pain [30-33]. These instruments are examples rather than default choices. Pediatric populations and culturally or linguistically distinct settings should use validated age-, fracture/site-, language-, and setting-appropriate measures; adult-oriented instruments should not be used when their validity or interpretability is uncertain. Objective range of motion, strength, return to activity, radiographic alignment or union, treatment conversion, and resource use can be added where clinically relevant.

Outcome timing is equally important. A single endpoint cannot describe the recovery trajectory of a fracture intervention. Early assessments may detect pain, swelling, neurovascular problems, skin injury, or loss of reduction; intermediate assessments may capture functional recovery and delayed union; longer follow-up may be needed for malunion, nonunion, persistent disability, avascular necrosis in selected fractures, or reoperation. The follow-up schedule should be justified by the expected biology and clinical course rather than chosen solely for convenience.

Recent research demonstrates the feasibility of repeated functional assessment in traditional fracture-care studies [14]. Future studies should use repeated measures intentionally so that the timing of recovery, treatment modification, and harms can be interpreted longitudinally rather than from a single late endpoint.

Core analytical principles and context-dependent advanced methods

Descriptive studies remain useful for identifying practice patterns and generating safety hypotheses, but comparative claims require effect estimates, uncertainty intervals, and an analysis strategy aligned with the design. Core principles are to preserve randomized comparisons when randomization is used; measure key prognostic factors before treatment; prespecify confounders from clinical knowledge and a causal model rather than univariable P-values; use longitudinal methods that reflect the repeated-measures structure; and report the amount, timing, and handling of missing data transparently. Propensity-score weighting or matching, doubly robust estimation, instrumental-variable analysis, and formal missing-not-at-random sensitivity analyses are context-dependent advanced options, not universal requirements. They should be used only when study objectives, sample size, overlap, data quality, and identifying assumptions make them defensible [23].

For repeated outcomes, mixed-effects models or generalized estimating equations are often appropriate because they account for within-person correlation; time-to-event methods may be preferable for union, complications, treatment conversion, or reoperation. Model form should match the clinical recovery trajectory and be prespecified where possible. Missingness should be reported by group and time point, with the primary approach justified. Maximum-likelihood models or multiple imputation can be used under plausible missing-at-random assumptions, while more advanced sensitivity analyses for departures from those assumptions should be reserved for settings in which they are informative and feasible. A prespecified primary outcome and time point should be distinguished from secondary or exploratory analyses, with multiplicity control used when the inferential objective requires it.

Safety surveillance

Safety surveillance is the domain in which the current evidence is both most clinically compelling and most methodologically vulnerable. Severe complications are repeatedly described in hospital-based series and systematic reviews [7-12]. These data justify concern but do not provide a complete denominator for risk, because uncomplicated patients who never attend hospital are often invisible to the study. Conversely, relying only on passive reporting may miss non-catastrophic harms such as skin injury, prolonged stiffness, sensory symptoms, minor loss of reduction, or treatment dissatisfaction.

Future prospective studies should prespecify adverse events before enrollment and use active surveillance at scheduled contacts. Events should be defined clinically and temporally, with severity and relationship to the intervention documented where possible. Relevant fracture-specific events include loss of reduction, pressure or skin injury, neurovascular compromise, compartment syndrome, infection, delayed union, nonunion, malunion, refracture, treatment conversion, hospitalization, surgery, amputation, and death. The phrase "no complications were observed" should be reserved for studies that actually sought complications systematically.

Active surveillance also improves attribution. If the intervention is multi-component, researchers should record which component was being delivered when an event occurred, whether the protocol was followed, and whether the event was related to manipulation, splinting, topical treatment, mobilization, or another aspect of care.

Process evaluation and context

A treatment can fail because its underlying mechanism is ineffective or because it was not delivered, received, or implemented as intended. Process evaluation is therefore especially important for TBS. Studies should measure treatment fidelity, patient adherence, practitioner adaptation, co-interventions, referral decisions, and contextual factors that influence delivery. Complex-intervention guidance emphasizes that context and implementation are part of the causal system rather than background noise [2,17,18].

Context should also be treated respectfully and analytically. Traditional healers may provide geographic access, flexible payment, culturally trusted communication, and continuity within communities. Qualitative work in Tanzania and recent expert consensus from East and West Africa have highlighted both unsafe practices and opportunities for referral, training, and collaboration [19,24]. The research question should therefore be framed around patient outcomes and safe care pathways, not around a binary contest between "traditional" and "modern" medicine. Table 2 sets out the core intervention description that follows from these considerations.

**Table 2:** Proposed TBS-8 methodological framework for evaluating traditional bonesetting TBS: traditional bonesetting.

Domain	Core question	Proposed core design / reporting action	Common pitfall
1. Intervention specification	What exactly was delivered?	Component-level description of materials, procedures, dose, timing, tailoring, modifications, and fidelity	Treating “TBS” as a single exposure without component-level definition
2. Provider characterization, training, and expertise	Who delivered the intervention, and how was expertise acquired or recognized?	Training pathway or source of expertise, years of experience, case volume, number of practitioners, certification where applicable, and study-specific preparation	Using “experienced bonesetter” without operationalizing training or expertise
3. Comparator definition	What did the comparison group actually receive?	Equivalent protocol description, provider details, timing, rehabilitation, crossover and escalation rules	Comparing TBS with an undefined “hospital” or “usual care” group
4. Baseline prognostic measurement	Were groups clinically comparable before treatment?	Pretreatment pain/function, fracture severity, time from injury, comorbidities, access, and preference variables	First measuring severity only after TBS exposure or after complications develop
5. Allocation and confounding control	Can the study support the intended causal comparison?	Randomization only when ethical and equipoise exists; otherwise, prespecified causal model and confounder control, with overlap assessment when relevant	Randomizing urgent surgical indications, selecting confounders by P-value, or using advanced methods without defensible assumptions
6. Outcome and follow-up architecture	Are recovery and clinically meaningful outcomes measured at appropriate times?	Prespecified primary outcome/time point, validated age- and setting-appropriate measures, repeated follow-up, and effect estimates with confidence intervals	Using adult-oriented or unvalidated scales by default, a single late endpoint, or no pretreatment outcome
7. Active safety surveillance	Were harms actively sought, classified, and attributed?	Prespecified adverse-event definitions, scheduled ascertainment, severity grading, serious-event and conversion reporting	Interpreting “no complications reported” as evidence of safety after passive follow-up
8. Process evaluation	Was the intervention delivered and received as intended?	Fidelity, adherence, deviations, co-interventions, referrals, crossovers, and contextual factors	Attributing null or harmful outcomes to the intervention without checking implementation

The TBS-8 framework for future clinical research

**Figure 1:**
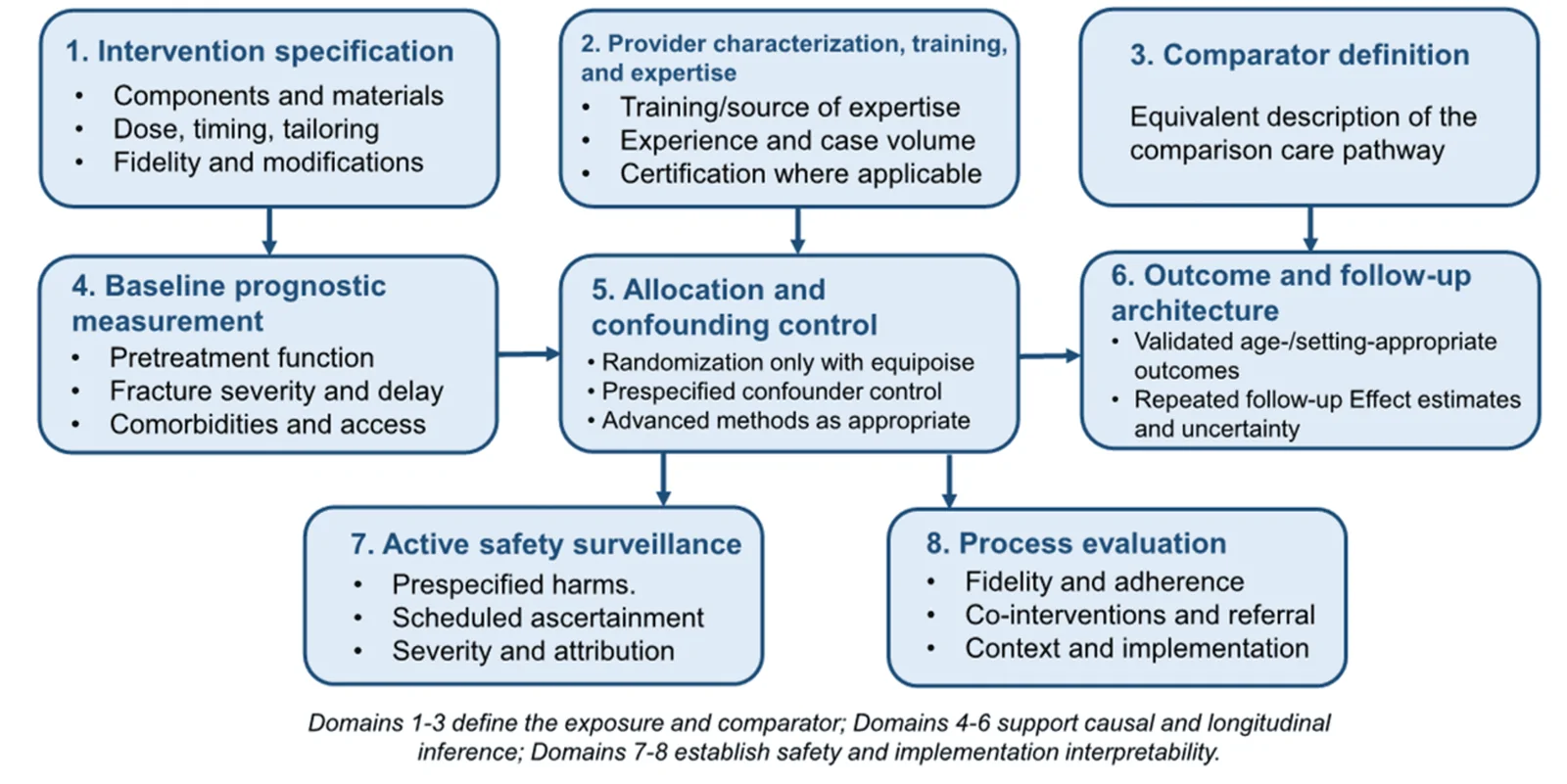
TBS-8 methodological framework for traditional bonesetting research The framework organizes eight proposed core methodological domains intended to support reproducibility, comparability, longitudinal interpretability, and safety-conscious research. It is conceptual and requires external consensus and prospective validation; it is not a validated reporting checklist or treatment guideline.

The recurring problems described above can be translated into the TBS-8 framework, an eight-domain conceptual model for future TBS research (Table 3 and Figure 1).

**Table 3:** Core TIDieR-informed intervention description for traditional bonesetting studies TBS: traditional bonesetting; TIDieR: Template for Intervention Description and Replication.

Domain	Core information to report	Why it matters
Intervention rationale	Clinical indication and intended role of each component	Separates testable clinical logic from cultural or historical description
Manipulation / reduction	Technique, analgesia, imaging, number of attempts, reduction criteria, post-reduction assessment	Permits replication and assessment of procedural risk
External support	Material, padding, limb position, tightness or pressure approach, joints included, duration, adjustment schedule	Mechanical behavior and pressure-related harms depend on application details
Topical or herbal treatment	Ingredients, preparation, dose, frequency, sourcing or quality control, skin monitoring	Allows reproducibility and attribution of benefits or adverse effects
Manual treatment / massage	Technique, anatomical target, session duration, frequency, timing in relation to fracture healing	Prevents vague descriptions such as "massage was given"
Mobilization / rehabilitation	Start time, exercise type, progression, supervision, home program, adherence	Recovery can depend on mobilization dose as much as on immobilization
Provider	Training pathway or source of expertise/recognition, years of experience, case volume, number of practitioners, formal certification where applicable	Provider characterization permits examination of expertise-related outcome heterogeneity
Tailoring and modification	Criteria for changing splint, activity, topical treatment, or referral	Clarifies whether individualized treatment is protocolized or discretionary
Fidelity	How delivery was monitored and deviations recorded	Distinguishes intervention failure from implementation failure
Comparator	Same level of detail as the TBS arm	Prevents invalid comparison against an undefined "usual care" group

The framework is not a validated reporting checklist, clinical guideline, or consensus standard. Rather, it proposes eight core methodological domains for consideration before claims about effectiveness, safety, or implementation are interpreted. These domains are intended as a TBS-specific translational scaffold, not as mandatory minimum requirements. External consensus work and prospective validation are needed before the framework can be treated as a formal reporting standard.

The ordering is deliberate. Domains one to three define the exposure, provider, and comparator; domains four to six address pretreatment prognosis, allocation/confounding, and longitudinal outcome measurement; and domains seven and eight address active safety surveillance and implementation. The framework is intended as a practical organizing scaffold, not a hierarchy or mandatory checklist. Figure 1 summarizes these relationships.

Choosing the study design

**Figure 2:**
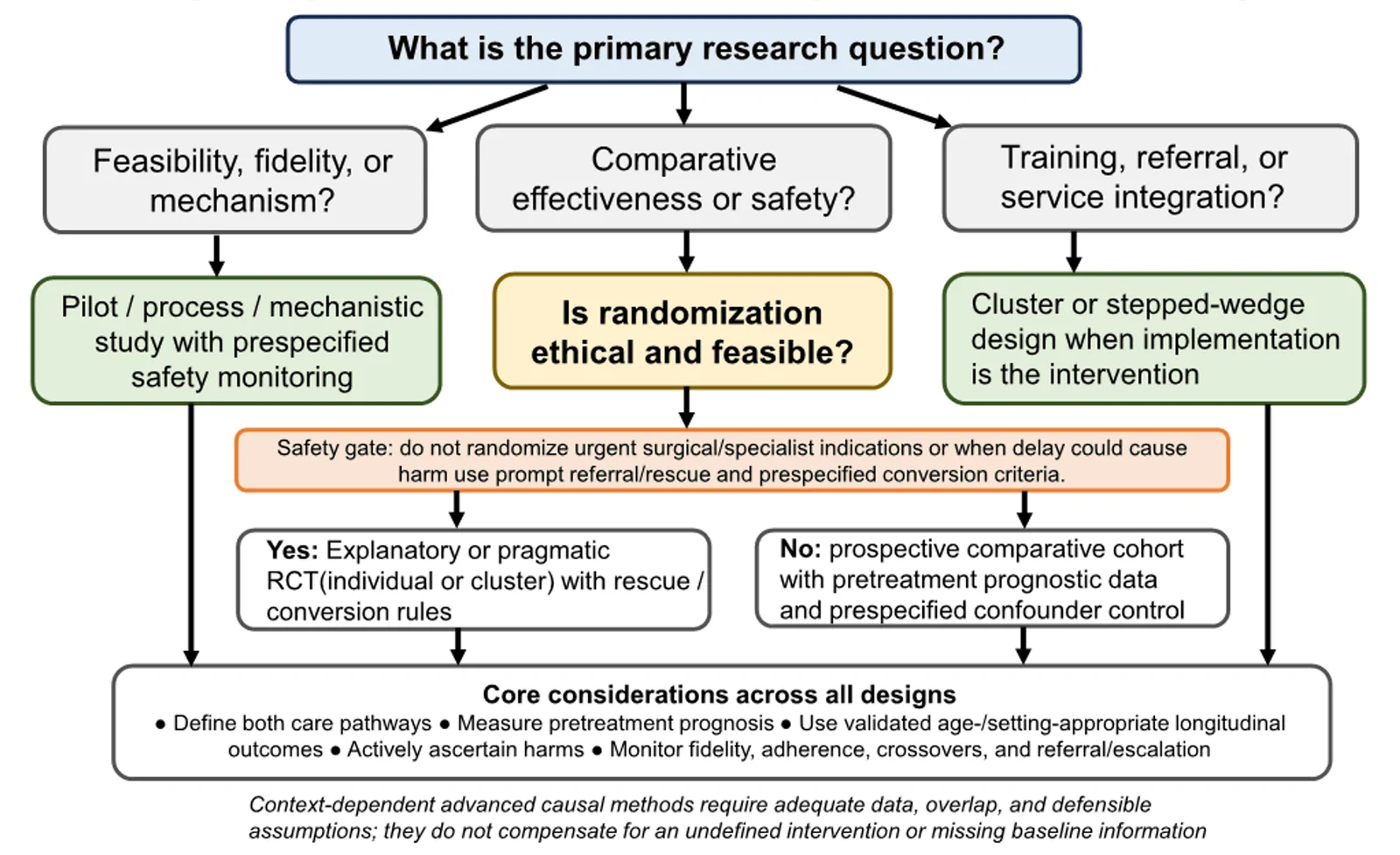
Study-design decision aid for evaluating traditional bonesetting Design choice should follow the research question, clinical equipoise, safety, and feasibility. Injuries with established urgent surgical or specialist indications should be excluded from randomization and referred promptly. Across designs, core considerations are clear intervention/comparator definition, pretreatment prognostic measurement, validated age- and setting-appropriate longitudinal outcomes, active safety surveillance, and process evaluation; advanced causal methods are context-dependent.

No single design is appropriate for every TBS question, and the TBS-8 framework should be applied to the research objective rather than used as a hierarchy. Feasibility, mechanistic, or fidelity questions may require pilot or process studies. Comparative-effectiveness questions may be addressed with randomization only when genuine equipoise exists, no urgent surgical or specialist indication is being deferred, and prespecified rescue/referral safeguards are in place. Pragmatic trials may be useful when the intervention is delivered in routine care [22]. Training, referral, or health-service integration may be better tested with cluster-randomized or stepped-wedge designs, as illustrated by emerging collaborative-care research [28]. Figure 2 provides a practical design decision aid.

Where randomization is not feasible or ethical, a prospective comparative cohort can still be informative if treatment groups are defined at initiation, baseline prognostic variables are measured before treatment, treatment preference and access variables are captured, overlap is evaluated where relevant, and analysis adjusts for prespecified confounders. The target estimand should be stated explicitly, and crossovers or treatment modifications should be recorded as part of the care pathway rather than hidden as protocol noise [23]. Advanced causal methods are optional and useful only when their assumptions match the data-generating process.

Design choice is also constrained by research governance. Protocols should define clinical equipoise, eligibility, withdrawal and treatment-conversion criteria, urgent referral and rescue triggers, who monitors serious adverse events, and how participants retain access to indicated hospital or specialist care. Injuries requiring urgent surgical or specialist management should not be randomized to a pathway that could delay indicated care. When traditional practitioners are research partners, investigators should also address informed consent, remuneration, professional liability, data ownership, authorship or acknowledgement, and mechanisms for resolving disagreement [19,24].

One of the most important improvements over much of the existing literature would be to enroll patients at the start of the treatment pathway rather than after complications occur. Starting observation before or at treatment initiation allows measurement of baseline severity, treatment components, modifications, adherence, outcomes, and harms in a defined denominator. Figure 2 emphasizes that these core considerations apply regardless of whether the final design is randomized, pragmatic, or observational.

Ethical and governance considerations

TBS research involves ethical questions beyond conventional informed consent. Strong cultural preference can constrain genuine equipoise, and referral to hospital may carry financial, geographic, or social costs. Nevertheless, participant safety takes precedence over study-group assignment: randomization to a TBS pathway is not appropriate when an injury has an established urgent surgical or specialist indication or when delay could cause harm. Protocols should explain alternatives in culturally appropriate language and prespecify urgent-referral, rescue, crossover, and treatment-conversion criteria. Research partnerships should also address power imbalances between licensed clinicians and traditional practitioners. The scientific goal should be to measure and improve care without delegitimizing local practice or endorsing unsafe practice by association.

Research agenda

First, intervention taxonomy should precede large effectiveness trials. Researchers should document the actual TBS care pathways used in each region and identify which components are common, optional, or highly practitioner-specific. Modern Mongolian medicine studies show that traditional fracture care can be decomposed into measurable components and followed longitudinally [14]; similar component-level work is needed across other settings.

Second, safety studies need defined treatment denominators. Hospital complication series remain clinically valuable, but they should be complemented by prospective registries that enroll patients at TBS points of care and track outcomes regardless of later referral. This design would permit incidence estimates for prespecified adverse events, identify component-specific risk factors, and clarify the timing and reasons for treatment conversion. Linkage to referral hospitals can strengthen capture of serious events without assuming that every complication reaches formal care.

Third, practitioner training and referral pathways deserve direct evaluation. Evidence from Ghana shows that a structured training program can improve knowledge and selected practices and can establish active referral links [25]. A Nigerian feasibility study identified mutual willingness for formal training and collaboration [26], and Tanzanian studies have extended this work through collaborative training [27], a stepped-wedge cluster-randomized pilot of joint triage and treatment [28], and broader synthesis of intersectoral collaboration initiatives [29]. Future research should test clearly defined curricula, red-flag referral criteria, infection-control measures, splint-safety protocols, communication systems, and shared follow-up using patient-centered and safety outcomes. Integration should be staged, locally governed, and contingent on measurable safety performance rather than assumed to be beneficial because collaboration is culturally acceptable.

Fourth, future comparative studies should incorporate economic and access outcomes. TBS is partly sustained by cost, geography, trust, and convenience [6]. An intervention that improves radiographic outcomes but is inaccessible to the target population may have limited public-health impact. Conversely, affordability cannot justify preventable harm. Comparative research should therefore evaluate effectiveness, safety, cost, time to care, catastrophic expenditure, and patient experience together where relevant.

Finally, a TBS-specific reporting extension may eventually be useful, but it should be developed through formal consensus rather than by converting the TBS-8 framework directly into a checklist. Such work should include traditional practitioners, orthopedic clinicians, rehabilitation professionals, methodologists, patients, and health-system stakeholders. Consensus should address core intervention description, practitioner characteristics, common outcomes, active safety definitions, and referral/escalation criteria.

Implementation pathways

Implementation research should move from informal coexistence toward explicit, testable care interfaces. Potential models include joint training, standardized referral triggers, shared documentation, scheduled feedback between traditional practitioners and orthopedic services, and regional safety registries. Recent collaboration studies suggest that such interfaces are feasible in some settings [27-29], but implementation outcomes should include reach, adoption, fidelity, time to referral, patient experience, equity, and unintended harms. Local ministries, professional bodies, community representatives, and practitioner groups should participate in governance so that integration does not outpace evidence of safety or capacity.

Strengths and limitations of this review

This review addresses a methodological gap that is often obscured by debates about whether TBS is beneficial or harmful. Its principal strength is the integration of established complex-intervention and comparative-effectiveness principles with recurring design problems in the TBS literature, including provider heterogeneity, poorly defined comparators, confounding by indication, incomplete treatment denominators, and passive harm ascertainment. The TBS-8 framework is intentionally applicable across randomized, pragmatic, implementation, and observational research, but it remains a proposed conceptual tool. This was a purposive rather than protocol-driven systematic review: source selection was iterative, exact study-set reproducibility is therefore limited, and examples may favor sources that illustrate the argument. The review cannot estimate the prevalence of methodological deficiencies or pooled effectiveness, and relevant non-English or non-indexed studies may have been missed. The framework has not undergone Delphi consensus, external validation, or prospective testing and should be evaluated across settings, age groups, and fracture types before formal checklist development.

## Conclusions

TBS should not be evaluated as a single, uniform treatment. It is a family of complex, practitioner-dependent fracture-care pathways that may combine manipulation, external support, topical treatment, manual therapy, immobilization, mobilization, rehabilitation, and referral decisions. Credible research must define those components and providers before comparing outcomes; measure pretreatment prognosis; address treatment allocation and confounding; use validated age- and setting-appropriate longitudinal outcomes; actively seek harms; and document fidelity and context. The TBS-8 framework organizes these considerations into eight proposed core domains for future study design and reporting.

The goal is neither to validate nor dismiss TBS by label. It is to replace vague exposure categories and retrospective complication capture with reproducible care pathways, defined denominators, transparent causal assumptions, and patient-centered safety monitoring. Recent training and collaborative-care studies indicate that provider behavior and referral systems can themselves be evaluated as modifiable interventions. Prospective testing and refinement of the TBS-8 framework should determine whether it improves the interpretability, comparability, and safety relevance of future research.

## Appendices

Literature identification strategy

Review Purpose

This was a purposive methodological literature review rather than a systematic or scoping review. The objective was to identify clinically and methodologically informative sources relevant to the design, measurement, safety, analysis, and implementation of traditional bonesetting research. No PRISMA screening denominator or exhaustive effect synthesis was intended.

Information Sources

PubMed was the principal biomedical database. Google Scholar, backward and forward citation chaining, and targeted searches of methodological literature were used as supplementary discovery methods. Zotero was used for reference organization. The final targeted literature update was completed on August 31, 2026.

Core Clinical PubMed Concept

The principal clinical search combined traditional bonesetting terminology with fracture and musculoskeletal-injury terminology. An example reproducible string is shown below:

("traditional bone setter"[Title/Abstract] OR "traditional bone setters"[Title/Abstract] OR "traditional bonesetter"[Title/Abstract] OR "traditional bonesetters"[Title/Abstract] OR "traditional bone setting"[Title/Abstract] OR "traditional bonesetting"[Title/Abstract]) AND (fracture*[Title/Abstract] OR "musculoskeletal injury"[Title/Abstract] OR "musculoskeletal injuries"[Title/Abstract])

Targeted Supplementary Searches

Additional searches combined traditional bonesetting terms with training, referral, collaboration, integration, complications, amputation, adverse events, safety, provider practice, and patient preference. Methodological searches targeted complex interventions, TIDieR, pragmatic trials, comparative effectiveness, observational causal inference, longitudinal mixed-effects models, treatment fidelity, and active adverse-event surveillance in orthopedic or fracture research.

Source Prioritization

Priority was given to peer-reviewed primary clinical studies, systematic reviews of TBS complications, studies of practitioner training or TBS-formal care collaboration, and established methodological guidance. Preprints and non-indexed material were not used as primary clinical evidence when a citable peer-reviewed source was available.

Selection and Synthesis

Source selection was purposive and iterative. Studies were included when they materially informed at least one methodological domain of the review. No formal risk-of-bias tool was applied because the purpose was not to estimate pooled treatment effects. Evidence was synthesized narratively to identify recurrent design, reporting, safety, and implementation problems and to inform the conceptual TBS-8 Framework.

Transparency Limitation

Because the review was not designed as a systematic evidence synthesis, this search description supports transparency and reproducibility of the literature-identification approach but should not be interpreted as proof of exhaustive study capture.
